# Atomistic Insights into the Tunable Transition from Cavitation to Crazing in Diamond Nanothread-Reinforced Polymer Composites

**DOI:** 10.34133/2020/7815462

**Published:** 2020-04-28

**Authors:** Lu-Wen Zhang, Wei-Ming Ji, Yue Hu, Kim Meow Liew

**Affiliations:** ^1^Department of Engineering Mechanics, School of Naval Architecture, Ocean and Civil Engineering, Shanghai 2002240, Shanghai Jiao Tong University, China; ^2^Department of Architecture and Civil Engineering, City University of Hong Kong, Kowloon, Hong Kong SAR, China

## Abstract

Cavitation and crazing in thermosetting polymers can be sophisticatedly designed for valuable applications in optics, electronics, and biotechnology. It is a great challenge for numerical study to describe the formations of cavity and fibrils in polymer composite due to the complicated interfacial interaction. To explore this challenging task, we exploit a two-phase coarse-grained framework which serves as an efficient atomistic level-consistent approach to expose and predict the transition between cavitation and crazing in a polymeric system. The coarse-grained framework is utilized to transmit the information between single phase and interface in polymer composite, and the learning tasks of force field are fulfilled through parameterization of mechanical performances and structural characterizations. We elaborate on the intrinsic characteristics of the cavitation-crazing transition in diamond nanothread- (DNT-) reinforced polymethyl methacrylate composites, in which DNT plays a specific role of nanomodulator to tune the cavity volume ratio. The transition from cavitation to crazing can be induced through a novel dissipative mechanism of opening an interlocked network, in which case the DNT is stretched to the aligned fibrils and links crazing tightly by interfacial adhesion. The designed computational framework can broaden the scope of theoretical tools for providing better insights into the microstructure design of polymer composites.

## 1. Introduction

The existences of cavitation and crazing are two unfavorable damage modes which contribute to the dynamic fracture of polymeric systems during the tensile deformation. Prior to crazing formation, the system undergoes a cavitation process, in which voids preferentially occur at transformation sites with a low local modulus [1–3], leading to “cavitation yielding” [4]. Crazing, originating from cavity nucleation, is the phenomenon of forming regional fibrils across the fracture plane [5–7]. Drawing of fibrils from the amorphous phase causes active dissipation of energy released from the crack tips, which is important for load-bearing applications [8].

Understanding the mechanisms behind these phenomena is of great significance in material sciences [9–14]. It can help applications in drug delivery using collapse cavitation [9], color printing using organized microfibrillation without ink [10], anisotropic conductivity using solvent crazing [11], and separator shutdown in lithium-ion batteries through thermal shrinkage of oriented fibrils [12]. However, harnessing of cavity and microfibril formation still remained as a challenging task due to the complicated interaction and competition between cavitation and crazing in the pure polymer systems.

The atomic insights and manipulation of cavitation and crazing in polymer nanocomposites have triggered a significant research interest. Analogous to the toughness mechanism of biological structural materials such as spider silk and abalone adhesive, mobile nanoparticles exhibit a novel dissipative mechanism to improve the toughness of polymer nanocomposites [15]. Utilizing the high mobility regions between the nanoparticles and the polymer chains, cavitation can be induced to the interface upon stretching, providing the functionality of tunable stiffness [16–18]. On the other hand, crazing can also be introduced to the nanocomposite by encapsulating nanoparticles, which can create “weak” points at the interface for craze initiation [19, 20]. During the crazing process, there are two important stages of nanoparticle rearrangement, which are alignment with the fibrils and expulsion from the fibrils [21]. A tunable transformation between cavitation and crazing could be achieved if the rearrangement process is well controlled, leading to a myriad of applications for polymer nanocomposites. However, this remains a difficult task due to the high mobility and uncertainty of nanoparticle motion during deformation.

Exploring the physical mechanisms behind cavitation and crazing using a computational approach is at great challenges. At the nanoscale level, although molecular dynamics (MD) is considered as a workhorse tool to provide reliable atomic insights in composite systems [22–24], large-scale atomistic simulation is still essential at the expense of computational efficiency, in order to cover the entire length of cavitation and crazing sites ranging from nanometer to micrometer. At the macroscale level, despite the fact that continuum mechanics can clearly display the cavitation and crazing phenomenon and characterize the crack-growth behaviors [25], the disentanglement of polymer chains that significantly influence the cavitation-crazing transition cannot be captured. To solve the drawbacks mentioned above and bridge the gap between nanoscale and macroscale, dissipative particle dynamics- (DPD-) based coarse-grained (CG) theory, which maintains lots of atomic details while overcoming the length- and time-scale limitations, has been applied [26–28]. The DPD method is more appropriate to study the problems of complex fluids, such as phase separation and material dispersion. The accuracy of CG models is strongly dependent on the parameterization of the interfacial interaction between nanoreinforcements and the polymer matrix and chain flexibility including chain scission and chain disentanglement. However, a robust CG technique for the representation of stress-strain response, interfacial interaction, cavitation, and crazing damages in polymer nanocomposites remains to be uncovered.

This work will explore the MD-based CG theory can be a promising tool to investigate the cavitation and crazing behaviors in polymeric systems while reaching a trade-off between computational efficiency and accuracy. Here, we propose a CG model which can successfully furnish a good correlation between the chain flexibility and the interfacial interaction. In addition, the mechanical properties and fracture behaviors are well examined by the results calculated from the MD model. It is revealed that the diamond nanothread (DNT), which is a one-dimensional ultrathin carbon-based nanomaterial [29–32], plays a role of nanoattractor for polymer chains, which can be utilized as a modulator to control the cavitation-crazing transition in polymer nanocomposites.

## 2. Results and Discussion

**Figure 1:**
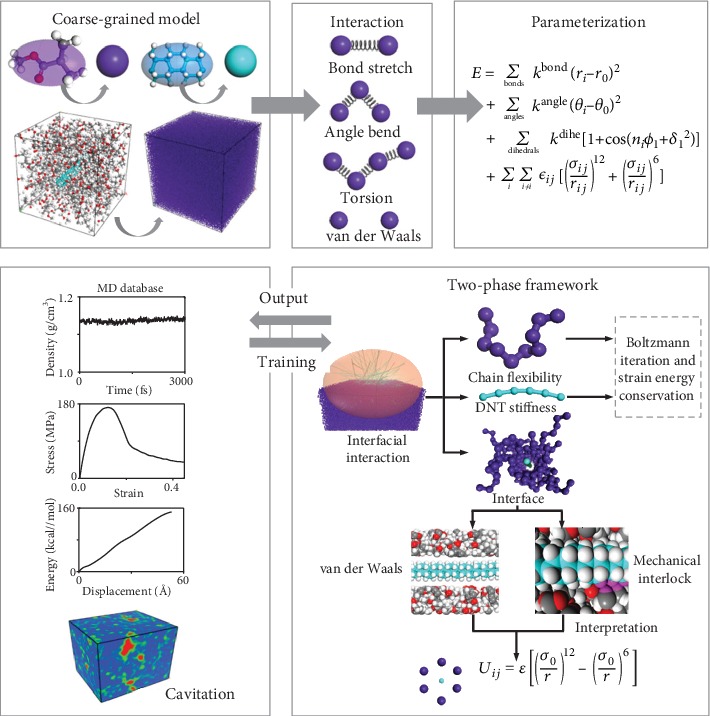
The coarse-grained model of PMMA filled with DNT and the computational graph for characterizing polymer chain flexibility and interfacial interaction.

We provide a physical insight into the transition between cavitation and crazing through the exploration of a novel simulation of the damage modes in polymer nanocomposites. The schematic diagrams of the atomistic and coarse-grained computational models of the DNT/PMMA hybrid material are presented in Figure 1. In the proposed CG model, the atomistic characteristics are well represented from the MD model, e.g., the chain flexibility of PMMA.

### 2.1. Simulation Mechanism

**Figure 2:**
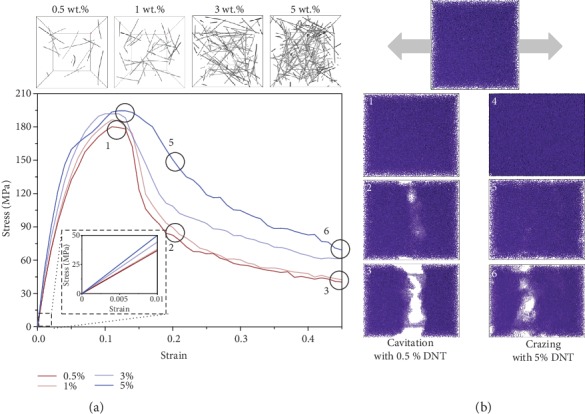
Effects of DNT content on stress-strain response of PMMA composites. (a) Stress-strain curves of PMMA composites reinforced with various weight ratios of DNT under tension; (b) cavitation and crazing phenomenon observed during deformation.

We begin by investigating how the cavitation and crazing form in the interior of DNT-filled PMMA composite under tensile deformation (Figure 2). The weight fraction ratios of the nanofillers are an essential factor in the computational modeling of hybrid nanocomposites; here, 0.5 wt.% to 5 wt.% DNTs are filled into PMMA matrix. The stress-strain curves related to varied spatial distribution are computed as illustrated in Figure 2(a). Apparently, an improved global mechanical performance of the nanocomposites, especially the fracture toughness, can be attained with the incorporation of DNT. In the process of tension, cavitation and crazing start occurring in the interior of the hybrid material, when the corresponding weight ratio is given as 0.5% and 5%, respectively, in Figure 2(b). In this study, the cavitation is characterized from density distribution function, and the voids in the three-dimensional density map are considered as cavities. The crazing is characterized directly from the geometric morphology in fracture surface. Here importantly, the higher ratio of the filler brings about a transition from cavitation to crazing.

**Figure 3:**
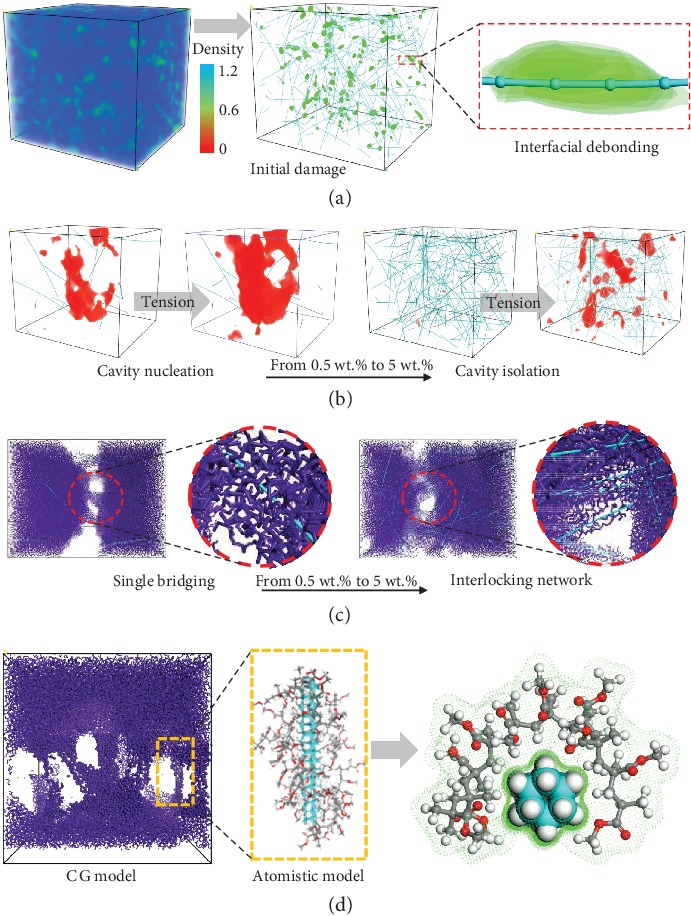
Cavitation and deformation pattern for hybrid nanocomposites. (a) Density distribution of polymer atoms in DNT/PMMA before onset of cavitation. Stripe shape in green color represents the slippage path of DNT reinforcement during tensile deformation. It is clearly observed that original damage initiates at the interface; (b) cavity (represented with red color) evolution with 0.5% and 5% DNT, respectively; (c) single-bridging and network interlocking in DNT/PMMA with 0.5% and 5% DNT, respectively; (d) crazes in DNT/PMMA modeling by the coarse-grained method and full molecular dynamics method. The Connolly surfaces (green dots) of the DNT and PMMA chains are depicted to present the nonbond interaction.

To provide insights into the physical origin of cavitation and reveal the mesoscale effects of the DNT filler, cavity revolution and deformation patterns are presented in Figure 3. Interfacial debonding is found to play a critical role in the cavitation resistance of the polymer composite. Prior to the onset of cavitation, interfacial debonding between DNT and the matrix occurs, as shown in Figure 3(a). The early interfacial debonding arises from the relatively weak interfacial shear stress with respect to the yield stress of polymer composites (see Supplementary Material Fig. S1). The interfacial debonding enables energy dissipation through van der Waals interactions and mechanical interlock [33, 34]. This could postpone storage of strain energy in transformation sites with low local modulus, leading to a larger yielding strain at a higher weight ratio of nanofillers because of the higher cavitation resistance in composites [35, 36]. The cavitation evolution further confirms the significant role of interfacial debonding in cavitation resistance. Following the interfacial debonding process, the growth of cavity is stabilized as the debonding sites increase with the increasing weight ratio from 0.5% to 5%, as shown in Figure 3(b). More significantly, a spatially homogeneous cavitation is detected in the scenario of 5 wt.% fillers, instead of the rapid nucleation of cavities in the amorphous phase, leading to a slower cavitation and smoother yielding process.

We discover that the local deformation of interlocking network formed by DNT can activate the change in the microvoiding mechanism between cavitation and debonding in polymeric systems. In terms of polymer composites without an interlocking network (0.5 wt.% DNT), cavitation is the predominant microvoiding mechanism. Free volumes/voids quickly enlarge at the unreinforced regions with low elastic modulus, and nanocracks appear on the void surfaces due to the growth and coalescence of cavities. In comparison, the prevalent microvoiding mechanism turns out to be debonding in polymer composites reinforced by an interlocking network (5 wt.% DNT). The void formations are homogeneously restrained within the network and prevented from coalescence. In this circumstance, debonding at the interface instead of internal cavitation is preferred. The DNT network ensures a better blocking of cavity nucleation as the cavities are forced to grow with a curved front between adjacent fillers, which is capable of inhibiting crack propagation (see Figure 3(c)).

We also identify a novel simulated mechanism “debonding-induced crazing” that controls the transition from cavitation to crazing in the polymeric system, which is attributed to the nanoattractor properties of DNT for polymer chain entanglement. The deformation patterns and frontier of the crack surface are presented in Figure 3(d). Results show that during the strain softening process, the interlocking network of DNT, which is located at the cavitation region will be gradually unlocked and turn to single bridging, and then, the DNT is able to align strongly with the tensile direction and absorb the polymer chain segments. The formation of regional crazes is explained by the specific characteristics of one-dimensional DNT, e.g., extremely thin diameter (around 0.5 nm [33]) and excellent interfacial interaction with the polymer matrix (174 kcal/mol [33]). As inelastic deformation proceeds, polymer chain entanglement on the surface of debonding DNT leads to regional crazes, and the craze thickens as the materials draw from being in an amorphous tangle into aligned fibrils.

We find that this novel simulated mechanism for the cavitation-crazing transition in polymeric systems is independent on the entanglement length of polymer chains. Commonly, the crazing performance strongly correlates with the length scale and rigidity of polymer chains [37]. If the number of monomers of the polymer chain exceeds the entanglement length, the mode of failure could change from cavitation to crazing. In this work, short polymer chains with 10 monomers are considered, whose feasibility has been well demonstrated in both atomistic modeling and coarse-grained modeling of PMMA composites [38–41]. The number of monomers per chain is less than the entanglement length needed for forming fibrils in this study, and crack propagation without crack fibrils across fracture plane (cavitation-induced damage) could be observed in the pure PMMA matrix (see Figure 3(d)). However, the DNT modulator successfully shifts the damage mode from cavitation to crazing. These findings signify that the simulated mechanism should be applicable to a wide range of polymeric systems.

### 2.2. Strain Rate-Controlled Strategy

**Figure 4:**
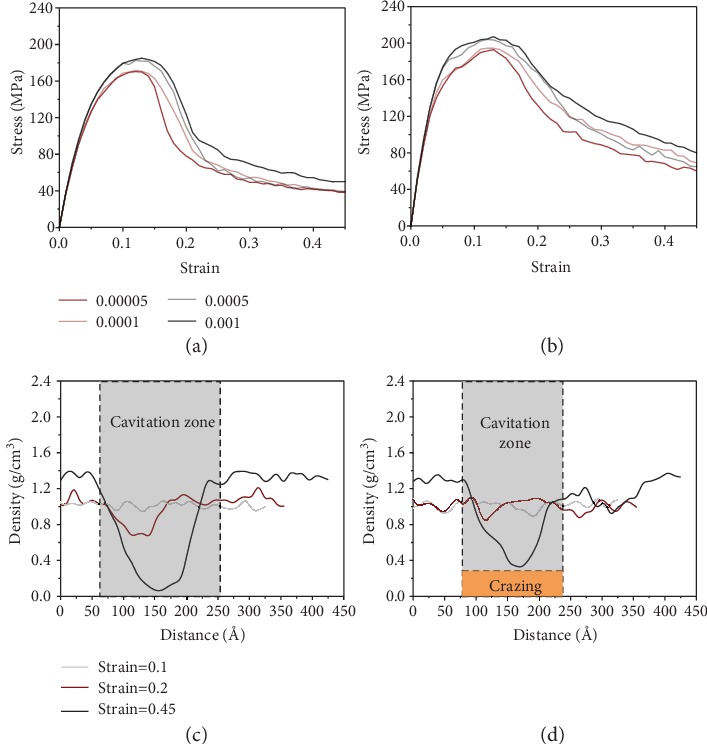
Effect of strain rate on stress-strain response of PMMA composites. (a) PMMA matrix under various tensile strain rates (per picosecond); (b) PMMA composites with 5 wt.% DNT weight fractions under various tensile strain rates (per picosecond); (c) concentration distribution of PMMA atoms at a strain rate of 0.00005; (d) concentration distribution of PMMA atoms at a strain rate of 0.001.

We investigate how the “debonding-induced crazing” responded to strain rate in the interior of DNT-reinforced PMMA composited under tensile deformation (Figure 4). Accounting for the strain rate applied to the polymeric system is an important factor affecting the mechanical response and failure mode of nanocomposites; strain rates ranging from 5 × 10^−5^/ps to 1 × 10^−3^/ps are studied in this work. The stress-strain curves obtained using different strain rates are plotted in Figures 4(a) and 4(b). Obviously, both the PMMA matrix and the PMMA composite exhibit strain rate sensitivity, as evidenced by rate-dependent Young's modulus, yield stress, and residue stress, which is consistent with previous findings of polymeric systems [42, 43]. It is noteworthy that a transition from cavitation-controlled damage to crazing-controlled damage is identified when the strain rate increases from 0.00005/ps to 0.001/ps, as shown in Figures 4(c) and 4(d).

**Figure 5:**
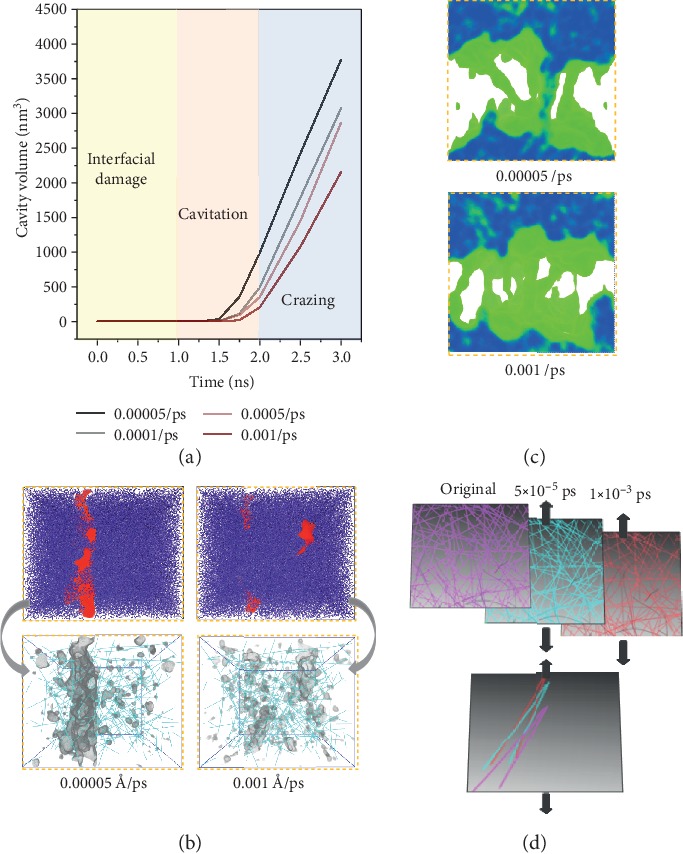
Cavity revolution and deformation patterns for polymer composites. (a) Cavity volume development at various strain rates. The interfacial damage stage is defined prior to the yielding point. The cavitation stage is defined as the nonlinear development of cavity volume. The crazing stage is defined as linear development of cavity volume. (b) Cavity distribution of PMMA composites with 5% DNT. The contour of crack tips that appeared on surfaces of cavities is represented with grey color. (c) Crazing pattern at various strain rates, and the fibrils are represented with green color. (d) DNT reorientation during tensile deformation.

We discover that “debonding-induced crazing” is rate dependent, which is explained by the variation in the degree of chain disentanglement with different strain rates. In general, the degree of polymer chain flexibility is strongly restricted at a higher strain rate; resistance of polymer chain alignment with the loading direction leads to enhancement of the mechanical properties of nanocomposites [44]. The lower-degree chain entanglement sites serve as regions for triggering craze initiation. The breakdown of crazes is strongly related to the disentanglement of polymer chains from fibrils during cavity nucleation. Short chains with higher chain flexibility in entanglement ensembles are able to slip away along the drawing direction, and this kind of disentanglement at regional fibrils is disadvantageous for a stable and sustained crazing process. Here, we find that chain disentanglement is less prevalent at higher strain rates, as evidenced by the cavity evolution in Figure 5(a). Results show that the growth rate of cavities decreases 30% as the strain rate increases from 5 × 10^−5^/ps to 1 × 10^−3^/ps, indicating that a lower degree of chain disentanglement occurs at a higher strain rate, which leads to activation of “debonding-induced crazing” in the polymeric system. As the chain disentanglement will be more prevalent at a lower strain rate, decreasing the strain rate from 5 × 10^−5^/ps will lead to similar cavitation damage in the DNT/PMMA composites, and the cavitation-to-crazing transition will not occur.

Chain disentanglement at various strain rates can also be characterized by the shear viscosity. Generally, the nanofillers are analogous to plasticizers in the polymeric system, and they are capable of reducing the viscosity of polymer [45, 46]. In this study, the shear viscosity is characterized through the contour of crack tips that appeared on the surfaces of cavities in Figure 5(b). The reduced shear viscosity is found to be more pronounced at a higher strain rate. Therefore, cracks have longer preferential initiating locations and onsets at multiple locations, delaying the coalescence of cavities and leading to “debonding-induced crazing,” as shown in Figure 5(c).

Moreover, the rate-dependent “debonding-induced crazing” can also be attributed to the local deformation of the interlocking network during crazing. Nanofillers are used to act as anchors for the disentanglement of polymer chains in the cavitation regions. The strain energy input into the polymeric system is dissipated through both chain disentanglement and local deformation of the nanofiller. We find a larger local alignment of nanofillers to the loading axis occurs at a higher strain rate, as evidenced by the two-dimensional projection of DNT distribution in nanocomposite in response to different strain rates presented in Figure 5(d). This kind of local alignment could help reorient and unlock the interlocked network, inducing the single bridging across the fracture plane. This novel dissipative mechanism will capture the strain energy needed for chain disentanglement, promoting the transition from cavitation to crazing. As a result, the thickened crazes at a higher strain rate lead to a larger degree of strain softening.

### 2.3. Spatial Distribution-Controlled Strategy

**Figure 6:**
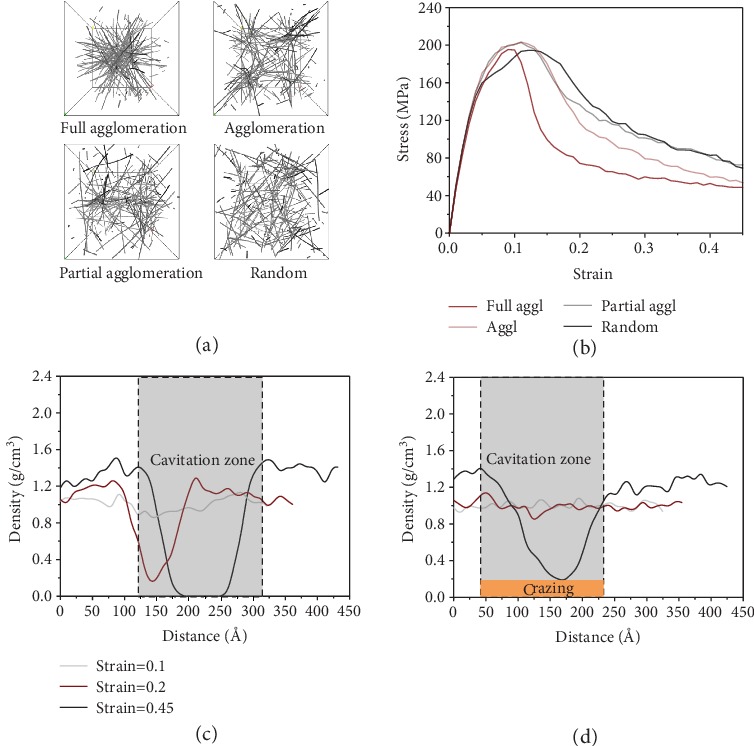
Effect of agglomeration on stress-strain response of PMMA composites. (a) Distribution of DNT with various agglomeration sizes; (b) stress-strain curves of the PMMA composites with various agglomeration sizes under tension; (c) concentration distribution of PMMA atoms at full agglomeration condition; (d) concentration distribution of PMMA atoms at random distribution condition.

We further investigate how the “debonding-induced crazing” responded to the spatial distribution of DNT in PMMA composite under tensile deformation (Figure 6). It has been conceded that nanofiller distribution is a critical factor affecting the mechanical performances of polymer nanocomposites. In this study, four kinds of distribution with 5 wt.% DNT are considered, and the agglomerated size follows an order of random distribution<partial agglomerated distribution<agglomerated distribution<fully agglomerated distribution. The representations of DNT distributions considered are shown in Figure 6(a). The stress-strain curves for the different agglomeration sizes are plotted in Figure 6(b). Obviously, nanofiller agglomeration has a detrimental effect on the mechanical properties of the PMMA composite, which is due to the heterogeneity brought by the agglomeration inside composites and stress concentration induced around the agglomerates [45]. It is important to realize a transition from cavitation to crazing is witnessed when the spatial distribution transforms from full agglomeration to random distribution, as shown in Figures 6(c) and 6(d).

**Figure 7:**
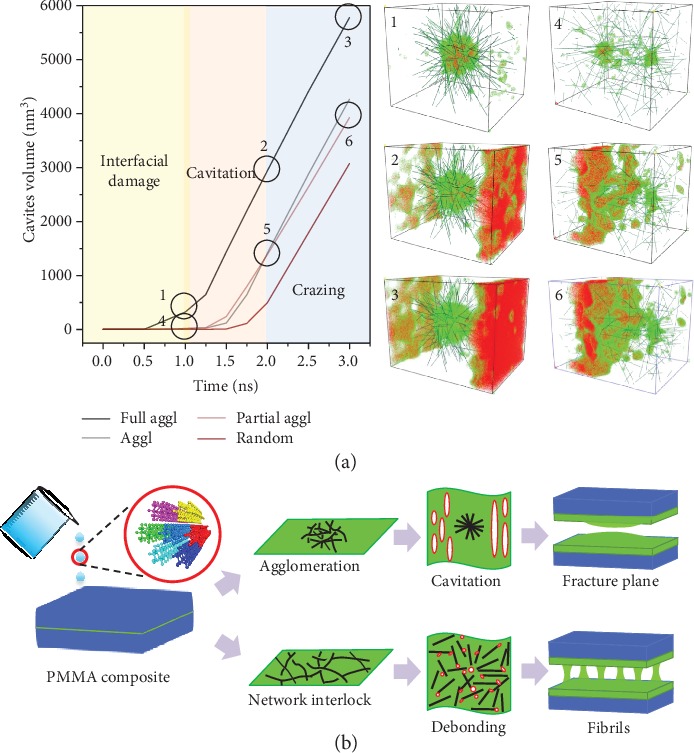
Cavity evolution and deformation patterns for polymer composites. (a) Cavity volume development at various strain rates and corresponding cavitation phenomenon. In the scenario of full agglomeration, fast cavity nucleation and fracture plane occurs early. In the scenario of partial agglomeration, cavities nucleate at the reinforced region and propagate along the interface; (b) schematic representation of microvoiding mechanism.

To provide insights into the physical origin of cavitation and reveal the mesoscale effects of DNT fillers, the cavity evolution and cavitation phenomena are presented in Figure 7. We find that there also exists a change of microvoiding mechanism through spatial distribution. As seen in Figure 7(a), onset of cavity initiation is gradually accelerated and cavity nucleation is more apparent when the agglomerated size is increased. The microvoiding mechanism changes from debonding to cavitation in a polymeric system, which is attributed to the geometrical constraints imposed to the network interlock.

We reveal that agglomeration promotes the disentanglement of polymer chains during crazing. Due to the heterogeneous deformation in the bulk matrix, chain disentanglement prefers to occur around the edges of agglomerates with low elastic modulus, causing early cavity nucleation. The easier chain disentanglement is mainly caused by the activation energy variation when DNT with a high aspect ratio and high stiffness is added to PMMA matrix. The activation energy in the reinforcing regions is greatly enhanced with the addition of DNT agglomerates. The strain energy is initially stored at the interphase whose activation energy is relatively lower. Due to unsatisfied load-transfer capacity between reinforcement and matrix regions, the strain energy prefers to transfer in the interphase rather than in the reinforced regions, which fails to create a back stress that inhibits coalescence of cavities [35]. Most of the strain energy is then dissipated by the disentanglement process, causing faster chain disentanglement and earlier break down of regional fibrils.

We find that “debonding-induced crazing” will disappear in the scenario of filler agglomeration, leading to a transition from crazing to cavitation in the polymeric system. This phenomenon can be explained by a geometrical constraint imposed on interlocking network, which can hinder the transformation from interlocking network to single bridging during the opening process. The cavity evolution shown in Figure 7(a) shows that the growth rate of cavities increases gradually with the increased agglomerated size. The cavitation patterns corresponding to various kinds of distribution are also provided. In the case of smaller agglomeration, cavitation tends to nucleate around the surface of reinforcement and expands along the tensile direction, during which a large local deformation is induced to the reinforcement network, leading to interlock opening and single bridging for craze formation. In contrast, in the case of larger agglomeration, cavitation induced by inhomogeneous deformation occurs in the unreinforced regions in which limited local deformation is imposed on the network reinforcement, rapidly resulting in cavitation failure. The schematic representation of the microvoiding mechanism is depicted in Figure 7(b). With the decrease of agglomerated size, the role of reinforcement turns from cluster with a filling effect to fiber with a bridging effect, and the transition from cavitation to crazing in the polymeric system is activated.

## 3. Conclusion

In conclusion, we propose a two-phase CG model which can efficiently represent the chain flexibility and interfacial interaction to reveal the intrinsic characteristic of tunable cavitation-crazing transition in DNT/PMMA composites. Owing to the ultrathin and excellent interfacial interaction with polymer chains, the DNT plays the role of nanoattractor for fibril formation during the debonding process, leading to “debonding-induced crazing.” It demonstrates that the cavitation-crazing transition can be manipulated by controlling the local deformation of the interlocking network, which is strongly dependent on the strain rate and spatial distribution. We discover that the cavity volume ratio in the DNT/PMMA composites can be controlled between 1% (crazing damage) and 10% (cavitation damage). These findings provide important insights into the bottom-up design of polymer nanocomposites and suggest that DNT is a promising candidate for advanced nanocomposites. The proposed two-phase CG framework could be applicable to a wide range of polymer composites and with an expectation that these findings can be utilized to achieve controllable induction of cavitation or crazing in polymer composite for desired applications.

## 4. Methods

The CG simulations are carried out based on the MD theory by using the Materials Studio 2017 software package. This part offers fundamental information about the computational methods and validation for the proposed CG model. For more details, we refer the reader to the information provided in the supplemental materials.

### 4.1. Model Construction

Using the coarse-grained method, the CG models which maintain a lot of atomistic details of the nanoscale systems are advantageous for solving complex physical problems [47, 48]. At the beginning, a PMMA polymer chain containing 10 monomers and a DNT reinforcement whose structural configuration is a hydronated CNT with chirality of (3, 0) are constructed. In a CG polymer model, a unit segment of methyl methacrylate (C_5_O_2_H_8_) is mapped into one bead with a total mass of 100.12 amu. For a CG DNT model, a unit segment of four atomic rings (C_24_H_24_) is mapped into one bead with a total mass of 312.46 amu.

A mesostructured template with different kinds of former types is built. In the scenario of random distribution, the PMMA beads and DNT beads are packed into the template with a dimension of 300 × 300 × 300 Å (*X* × *Y* × *Z*) using a Monte Carlo method with a predefined density of 1.1 g/cm^3^. In the scenario where fillers agglomerate, the template is filled with a slab former and a droplet former. DNT beads with predefined weight ratio are packed into a droplet former to represent the agglomeration condition. The remaining PMMA beads and DNT beads with an aspect ratio of 50 are randomly packed into the slab former to represent the random distribution condition. In this study, the CG models contain around 178300 beads, corresponding to a full atomistic model with around 2825900 atoms. The proposed CG method is found to greatly improve the computational efficiency. For example, it takes about 213600 seconds to obtain the results from MD simulation [33], while the time is reduced to 160 seconds in this work by using the same computing equipment.

### 4.2. Force Field Parameterization

The force field parameters used in the proposed CG models are obtained based on the strain energy conservation predicted from MD simulation [49–51] and examined by the Boltzmann inversion method and tensile stress-strain response. The force field is decomposed into four terms, the van der Waals interaction potential, bond potential, angle potential, and torsion potential.

The van der Waals interaction of the isolated coarse-grained beads is expressed by the Lennard-Jones (LJ) 6–12 potential to represent the nonbond interaction between PMMA beads and interfacial interaction between PMMA beads and DNT beads:

(1) $$\begin{array}{c} U_{ij}=\varepsilon \left[{\left(\frac{r_{0}}{r}\right)}^{12}-{\left(\frac{r_{0}}{r}\right)}^{6}\right], \end{array}$$

where *ε* denotes the equilibrium well depth and *r*_0_ denotes the equilibrium distance.

The elastic bond of two connected beads is expressed by the harmonic bond potential to give a measure of the mechanical stiffness of PMMA beads and DNT beads:

(2) $$\begin{array}{c} U_{b}=k_{b}{\left(r-r_{0}\right)}^{2}, \end{array}$$

where *k*_*b*_ is the bond elastic constant and *r*_0_ is the equilibrium bond length.

The bond angle between three connected bonds is expressed by the harmonic bond-angle potential to represent the chain flexibility of PMMA beads and DNT beads:

(3) $$\begin{array}{c} U_{\theta }=k_{\theta }{\left(\theta -\theta_{0}\right)}^{2}, \end{array}$$

where *k*_*θ*_ is the bending stiffness and *θ*_0_ is the equilibrium bond angle.

The dihedral angle between four connected bonds is expressed by the cosine potential to represent the chain flexibility of PMMA beads and DNT beads:

(4) $$\begin{array}{c} U_{d}=\frac{k_{d}}{2}{\left(1+\cos2\varphi \right)}^{2}, \end{array}$$

where *k*_*d*_ is the elastic constant of the dihedral angle and *φ* is the equilibrium dihedral angle.

The values of the CG parameter used in the present models are provided in Supplementary Material Table S2. We also examine the feasibility of the proposed CG model by comparing its results from the density evolution, interfacial shear stress between DNT and PMMA matrix, stress-strain relationship, and fracture phenomenon with those calculated from MD simulation. Results in supplementary material show that the proposed CG model can well predict the chain flexibility and mechanical behaviors of DNT-filled PMMA composites.

### 4.3. MD Simulation

The Condensed-phased-optimized Molecular Potential for Atomic Simulation Studies (COMPASS) is utilized in this work. The DNT is placed into a unit cell with a dimension of 30 × 30 × 50 Å (*X* × *Y* × *Z*). A periodic condition is applied to the unit cell. The PMMA polymer chains are randomly placed into the periodic box using the Monte Carlo method. A geometry optimization using the Smart algorithm is conducted to relax the atom positions in the polymer composites. After the minimization process, an annealing process is carried out with a constant volume and constant temperature (NVT) ensemble from 300 K to 800 K to further relax the structure. The system is then equilibrated under a constant pressure and constant temperature (NPT) ensemble at a temperature of 298 K and a pressure of 101 kPa with a time step of 1 fs. Finally, a constant-strain method is applied to study the stress-strain response of polymer composites under tensile loading. A series of expansions at a strain rate of 0.0001/ps are applied to the unit cell. Further details of the MD simulations can be referred to the previous study [33].

### 4.4. CG Simulation

The mesoscale molecules including PMMA beads and DNT beads with an aspect ratio of 50 are randomly placed into the periodic unit cell. Geometry optimization using the Smart algorithm is conducted to relax the atom positions in the polymer composites. After the minimization process, an annealing process is carried out under a NVT ensemble from 300 K to 800 K to further relax the structure. The system is then equilibrated under the NPT ensemble at a temperature of 298 K and a pressure of 101 kPa for 3 ns. The time step is set as 10 fs for the CG simulations. Finally, a constant-strain method is applied to study the stress-strain response of polymer composites under tensile loading. A series of expansions at a strain rate of 0.0001/ps are applied to the periodic cell along tensile direction. Preliminary results show that Young's modulus and ultimate stress of PMMA matrix in the CG simulations are calculated as 2.97 GPa and 170 MPa, which are in close agreement with the reported experimental results (2.86 GPa [38] and 167 MPa [52]).

## Data Availability

The data that support the findings of this study are available from the corresponding authors.

## Supplementary Materials

Supplementary Materials Figure S1: snapshots of DNT reinforcement pulling out from PMMA matrix. Figure S2: verification of the CG model using MD simulation. Figure S3: comparison of density between MD and CG simulations. Figure S4: structural deformation of PMMA bulk matrix at various strains. Figure S5: reinforcement reorientation during tensile formation. Figure S6: effects of long-chain length on the crazing of PMMA composite. Table S1: comparison of pull-out results calculated from MD and CG simulations. Table S2: parameter used in the CG model.
