# Supplementary material for: Atomistic Insights into the Tunable Transition from Cavitation to Crazing in Diamond Nanothread-Reinforced Polymer Composites
Source: Research (Wash D C). 2020 Apr 28;2020:7815462. doi: 10.34133/2020/7815462 (PMC7203679; doi:10.34133/2020/7815462)
Supplement: Supplementary Materials — Figure S1: snapshots of DNT reinforcement pulling out from PMMA matrix. Figure S2: verification of the CG model using MD simulation. Figure S3: comparison of density between MD and CG simulations. Figure S4: structural deformation of PMMA bulk matrix at various strains. Figure S5: reinforcement reorientation during tensile formation. Figure S6: effects of long-chain length on the crazing of PMMA composite. Table S1: comparison of pull-out results calculated from MD and CG simulations. Table S2: parameter used in the CG model. [file 7815462.f1.docx]

**1. Interfacial shear strength**

To study load transfer between the reinforcement and PMMA matrix quantitatively, interfacial shear strength ISS (τ) is estimated based on the change of non-covalent energy during the pull-out procedures. The pull-out processes are depicted in Fig. S1. The interfacial shear stress can be calculated as:

$=2E_{I}/DL^{2}$ (1)

where $E_{I}$ is the change of interfacial interaction energy, $L$ is the pull-out displacement which is about 45 Å, and D is the diameter of reinforcement which is about 4.44 Å.

(a)

| 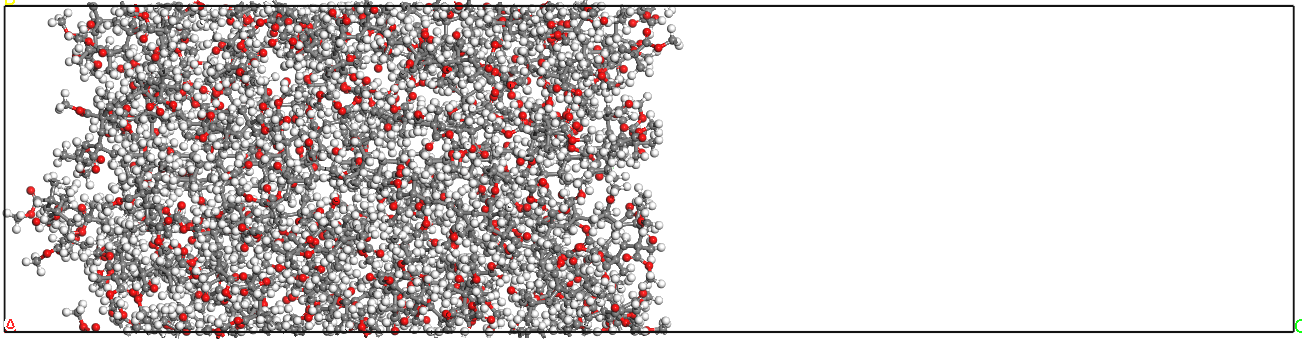  (b) | 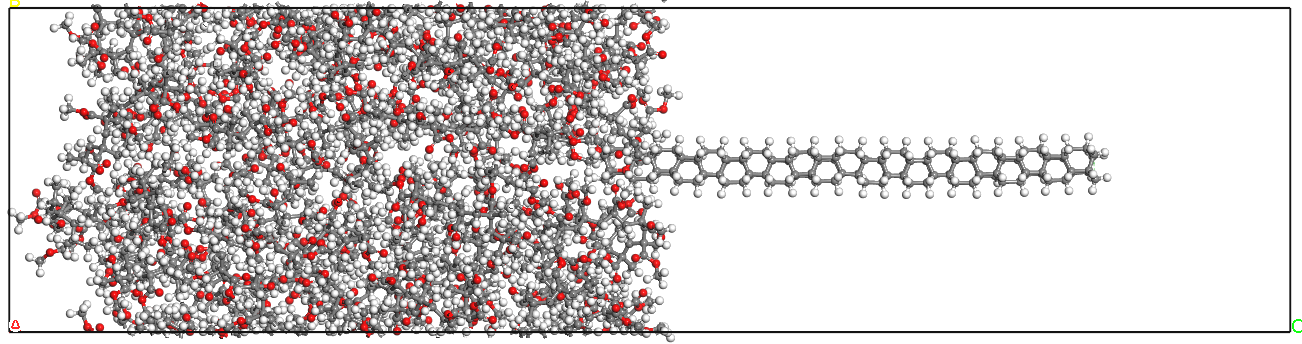 |
| --- | --- |
| 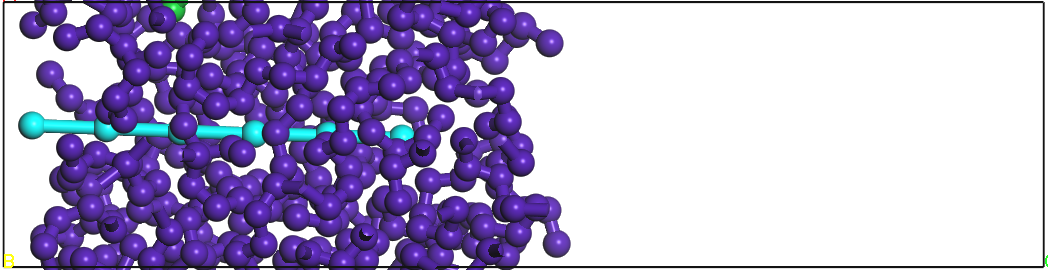 | 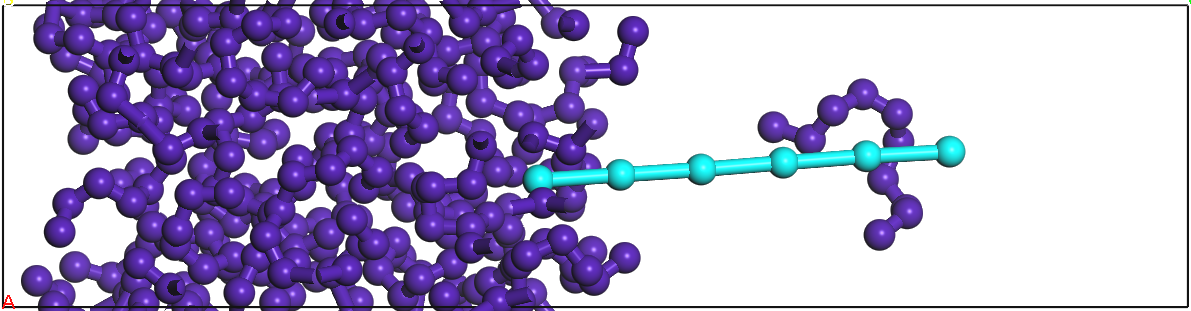 |
| **Fig. S1** Snapshots of DNT reinforcement pulling out from PMMA matrix. (a) MD simulations; (b) CG simulations. | |

**2. Validation of the CG models**

To validate our proposed CG models, the stress-strain responses together with the fracture phenomenon of PMMA matrix and DNT/PMMA composite predicted in this work are compared with those calculated by MD simulations. The cell dimensions of the CG structures were set as two times as large as the atomistic structure to reduce the uncertainty. The stress-strain curves of PMMA matrix and DNT/PMMA composite are shown in Fig. S1(a). As seen from the results, the mechanical properties of CG models agree well with those of atomistic models. For instance, the Young’s modulus of PMMA matrix and DNT/PMMA composite in the CG simulations are calculated as 2.97 GPa and 5.51 GPa, respectively, which are closed to our previous results from MD simulations (2.86 GPa for PMMA matrix and 5.31 GPa for DNT/PMMA composite [1]). Besides, we identify that the fracture phenomenon presented in the CG simulations are also comparable to that presented in the MD simulations, as seen Fig. S2(b). Based on the CG simulation results, continuous cracks propagating through the edge of PMMA matrix is observed, In terms of DNT/PMMA composite, the DNT reinforcement in the CG simulation is capable to block the crack propagation across PMMA composite, with some small cavities nucleating at the edge of the PMMA composite at the failure stage. These findings coincide with the failure behaviors of both bulk PMMA and DNT/PMMA composite predicted in MD simulations, indicating that the CG models proposed in this study are valid for predicting the mechanical properties of DNT/PMMA composites subjected to tensile loading. The comparison of density between MD and CG simulations is also depicted in Fig. S3. It is shown that the CG model can well reproduce the structural morphology comparing to MD model.

|   (b)  (a) | 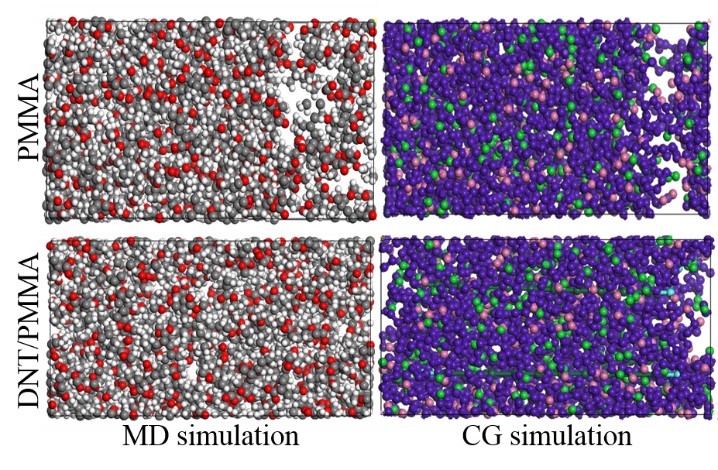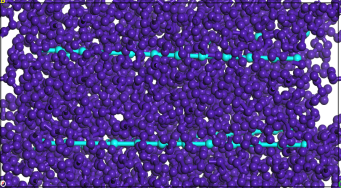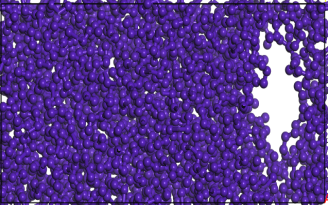 | |  |
| --- | --- | --- | --- |
| **Fig. S2** Verification of CG model using MD simulation. (a) Stress-strain curves of the PMMA matrix and DNT-C/PMMA composite under tension; (b) Fracture behavior of PMMA matrix and DNT/PMMA composite by means of MD simulation and CG simulation. | | |  |
| **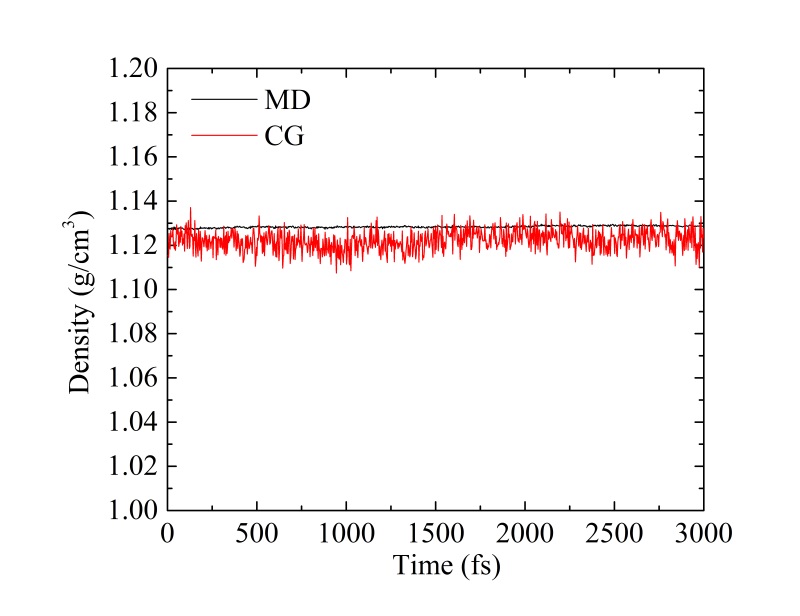**  (b)  (a) | | **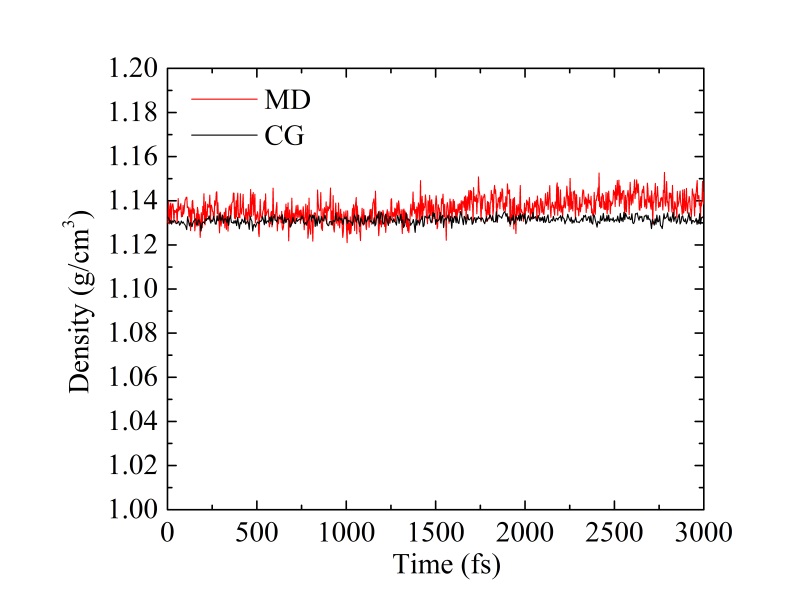** | |
| **Fig. S3** Comparison of density between MD and CG simulations. (a) PMMA matrix; (b) DNT/PMMA composite. | | | |

**3. Cavitation damage of pure PMMA matrix**

| 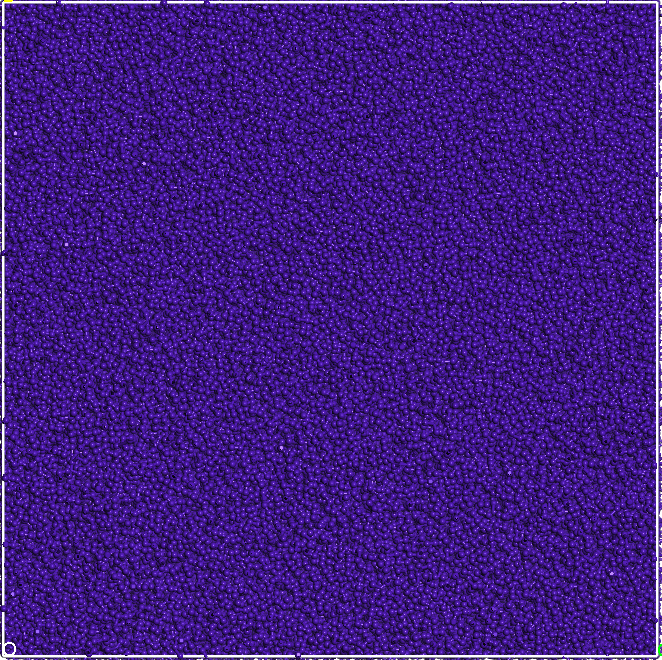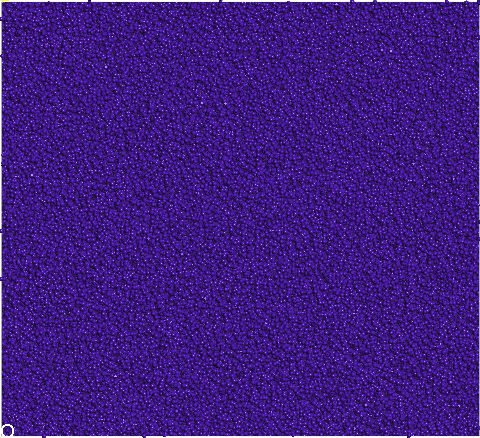 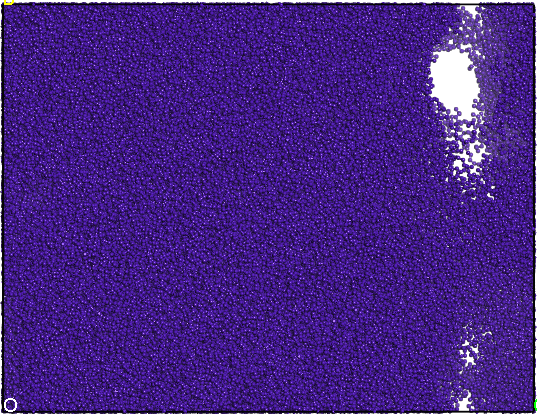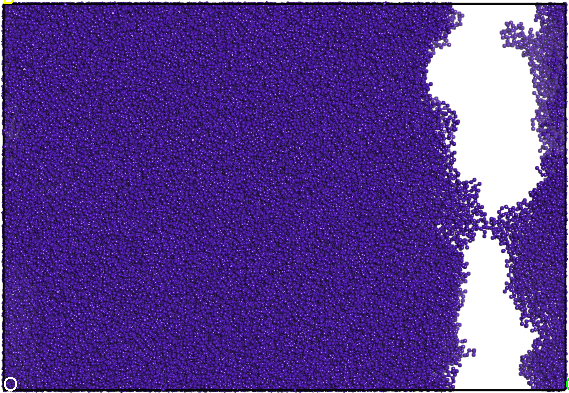  Strain=0 Strain=0.15 Strain=0.3 Strain=0.45 |
| --- |
| **Fig. S4** Structural deformation of PMMA bulk matrix at rarious strains. Cavitation failure rather than crazing is observed during tensile deformaiton. |

**4. Opening of interlocking network**

| **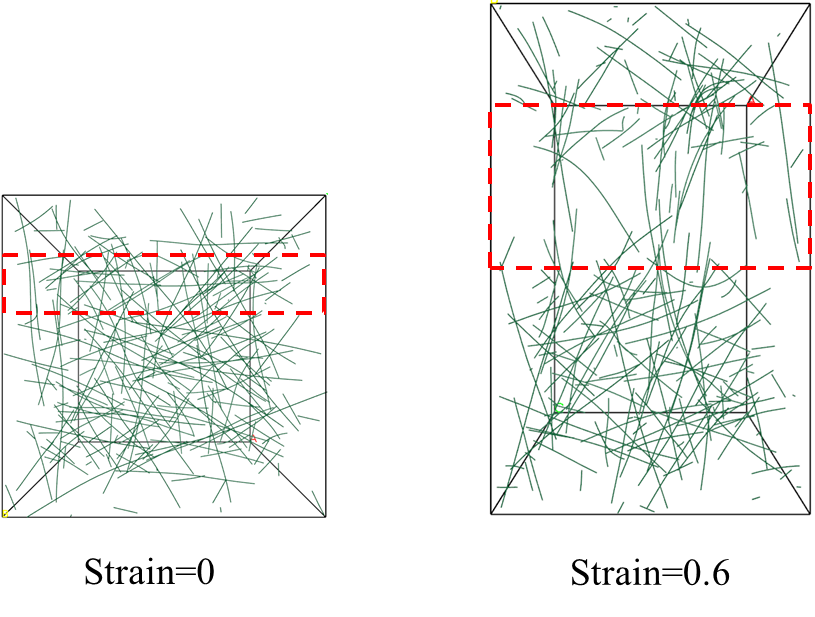**  Deformation |
| --- |
| **Fig. S5** Reinforcements reorientation during tensile deformation. The cavitation regions are marked with red rectangular. It can be observed that reinforcements located at the cavitation regions are able to reorient and align to the tensile direction during deformation. |

**5. Crazing damage in polymeric systems with long chain length**

| **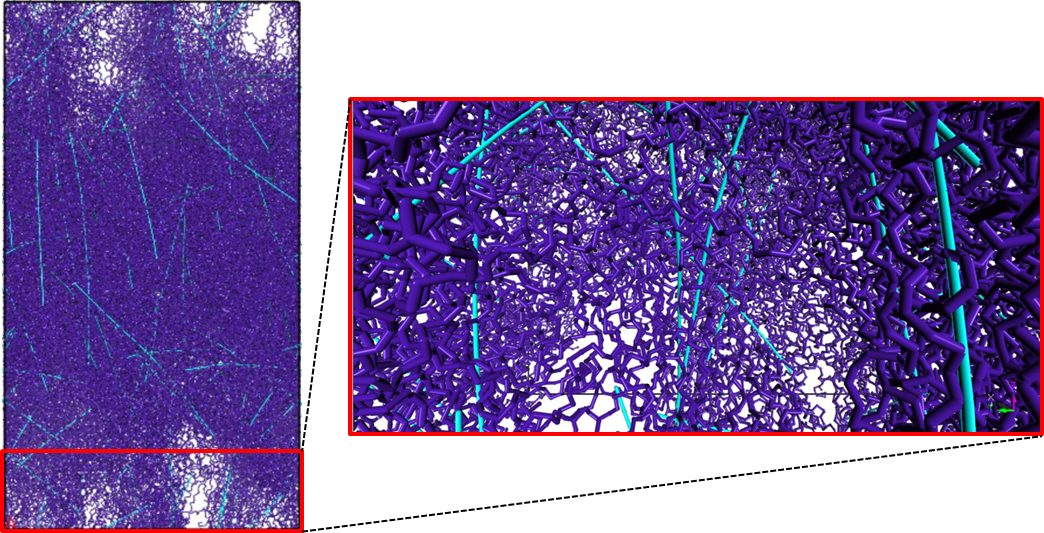** |
| --- |
| **Fig. S6** Effects of long chain length on the crazing of PMMA composite. It can be seen that DNT acts as nano-attractor, and polymer chain segments with chain length of 100 monomers absorb on the surface of DNT, resulting in fibrils across the fracture plane. The results provided confirmed proof that the discovered crazing-induced mechanism in this work is independent on the polymer chain length. |

Table S1 Comparison of pull-out results calculated from MD and CG simulations

|  | MD model | CG model |
| --- | --- | --- |
| Interfacial Interaction Energy | 135.45 kcal/mol | 205.73 kcal/mol |
| Interfacial Shear Strength | 66.69 MPa | 101.29 MPa |

**Table S2** Parameter used in the CG model

| Parameter | Description | Value | Unit |
| --- | --- | --- | --- |
| *σ* | Distance at zero energy between DNT beads | 6.51097 | Angstrom |
|  | Distance at zero energy between PMMA beads | 6.275 | Angstrom |
|  | Distance at zero energy between DNT and PMMA beads | 7.58 | Angstrom |
| *ε* | Depth of the potential well between DNT beads | 7.3 | Kcal/mol |
|  | Depth of the potential well between PMMA beads | 0.985 | Kcal/mol |
|  | Depth of the potential well between DNT and PMMA beads | 3.88 | Kcal/mol |
| *k_b_* | Elastic constant of DNT bond | 251.75 | Kcal/mol/Å^2^ |
|  | Elastic constant of PMMA bond | 164.61 | Kcal/mol/Å^2^ |
| *r_0_* | Equilibrium bond length of DNT bond | 8.5764 | Angstrom |
|  | Equilibrium bond length of PMMA bond | 4.02 | Angstrom |
| *k_θ_* | Bending stiffness of DNT | 10278.6 | Kcal/mol/Å^2^ |
|  | Bending stiffness of PMMA | 109.89 | Kcal/mol/Å^2^ |
| *θ*_0_ | Equilibrium bond angle of DNT | 180 | Degree |
|  | Equilibrium bond angle of PMMA | 112 | Degree |
| *k_d_* | Barrier height of PMMA | 40 | Kcal/mol |

**References**

**[1]** Zhang, L. W., Ji, W. M., & Liew, K. M. Mechanical properties of diamond nanothread reinforced polymer composites. Carbon 132, 232-240 (2018).
